# Complete genome sequence of a potential new species *Vibrio* sp. NTOU-M3 isolated from hard clam, *Meretrix taiwanica,* in Taiwan

**DOI:** 10.1128/mra.00810-24

**Published:** 2024-09-27

**Authors:** Che-Chun Chen, Wei-Hsiang Lin, Te-Hua Hsu, Ying-Ning Ho

**Affiliations:** 1 Doctoral Degree Program in Marine Biotechnology, National Taiwan Ocean University, Keelung, Taiwan; 2 Taiwan Oceans Genome Center, National Taiwan Ocean University, Keelung, Taiwan; 3 Institute of Marine Biology, National Taiwan Ocean University, Keelung, Taiwan; 4 Department of Aquaculture, National Taiwan Ocean University, Keelung, Taiwan; 5 Center of Excellence for the Oceans, National Taiwan Ocean University, Keelung, Taiwan; Montana State University, Bozeman, Montana, USA

**Keywords:** *Vibrio*, hard clam, *Meretrix taiwanica*, Oxford Nanopore Technologies

## Abstract

*Vibrio* sp. NTOU-*M3* is a potential new bacterium isolated from hard clam (*Meretrix taiwanica*) in the estuarine region of Taiwan. The complete sequences obtained using Oxford Nanopore Technologies and Illumina sequencing consist of a 3,272,438-bp large circular chromosome and a 1,584,497-bp small circular chromosome.

## ANNOUNCEMENT

The genus *Vibrio* consists of a group of marine bacteria that infect several genera of marine shellfish (1). The novel *Vibrio* sp. NTOU-M3 was isolated from hard clam (*Meretrix taiwanica*) obtained from Heping Island Fish Market, Keelung City, Taiwan, and sourced from the estuarine region of Taiwan. First, 0.1 g tissue was collected and mixed with 2 mL ddH_2_O, and then 100 µL mixture was added with 1/10 marine broth (1 mL). Finally, it was dropped on 1/10 marine broth agar plates (HiMedia Laboratorie, India). The plates were incubated at 37°C for 12 hours. A single colony purified by three rounds of streaking was selected for identification. Genomic DNA for both Illumina and Nanopore sequencing was extracted using the Nanobind CBB Kit (PacBio, USA). The DNA quantity was determined using the Qubit dsDNA BR assay kit (Thermo Scientific, USA). The 16S rRNA gene sequence was amplified by primers 27F and 1492R (2). Sanger sequencing yielded the nearly complete 16S rRNA gene sequence (PP981031). This 16S rRNA sequence was compared by NCBI BLASTn and limit to sequences from the type material. The most similar species is *Vibrio harveyi* NBRC 15634 (98.30%, NR_113784). The optimal identity thresholds for potential new species were ∼98.7% (3).

The Illumina sequencing library was generated using the TruSeq Nano DNA Library Prep Kit (Illumina), according to the manufacturer’s instructions. Genomic DNA was fragmented by sonication at 350 bp and then purified using Sample Purification Beads (Illumina). The NovaSeq X Plus platform was used by Genomics BioSci & Tech to generate 150-bp paired-end reads. Raw Illumina reads (total reads: 10,106,288) were trimmed using Trimmomatic v0.39 (4) and quality-controlled using fastp v0.23.4 (5). Long-read sequencing library was prepared using a the Nanopore genomic DNA ligation kit (SQK-LSK110) and sequenced using a Flongle flow cell R9.4.1 (FLO-FLG001) for 96 hours on the MinION platform. Nanopore data were basecalled using Dorado v0.6.0 (https://github.com/nanoporetech/dorado) and filtered by Nanofilt v2.8.0 (6) to generate Q12 data (raw reads: 50,476; N50: 32,611). Assembly was performed using Nanophase v0.2.3 (7) with the hybrid method, resulting in two circular chromosomes (average coverage: 243×) and rotated to start at the dnaA gene using the Circlator v1.5.5-docker5 (8). The genome includes two chromosomes of 4,856,935 bp with a G + C content of 43.8%. The final genome was annotated using DFAST v1.6.0 (9), revealing 4,279 CDSs, 28 rRNA genes, and 105 tRNA genes. The genome assembly had a completeness of 100% and contamination level of 0%, as confirmed by using CheckM v1.2.2 (10). The novel *Vibrio* sp. shows low ANI value (75.79%) and the dDDH (24%) to the closest type strain, *Vibrio harveyi* NBRC 15634 via CJ Bioscience’s online ANI calculator v0.91 (11) and TYGS v391 (12). In addition, antiSMASH 7.0 (13) analysis identified four secondary metabolite biosynthetic gene clusters, namely, ectoine, hserlactone, NRP-metallophore, and arylpolyene, in the large chromosome and three BGCs including a betalactone and two RiPP-like in the small chromosome of the *Vibrio* sp. NTOU-M3. The contributions of this complete genome sequence encourage further research into the relationship between *Vibrio* and hard clam *M. taiwanica*.

## Data Availability

Default parameters were used except where otherwise noted. This genome project has been deposited in the National Center for Biotechnology Information (NCBI) with Bioproject accession number PRJNA1136137 and BioSample accession number SAMN42503066. The raw reads are available at the NCBI Sequence Read Archive under accession number SRX25366312 to SRX25366313. The genome sequence can be found in GenBank under accession number CP162100 to CP162101.
